# Death-associated protein kinase 1 mediates Aβ42 aggregation-induced neuronal apoptosis and tau dysregulation in Alzheimer's disease

**DOI:** 10.7150/ijbs.66760

**Published:** 2022-01-01

**Authors:** Tao Zhang, Yongfang Xia, Li Hu, Dongmei Chen, Chen-Ling Gan, Long Wang, Yingxue Mei, Guihua Lan, Xindong Shui, Yuan Tian, Ruomeng Li, Mi Zhang, Tae Ho Lee

**Affiliations:** Fujian Key Laboratory of Translational Research in Cancer and Neurodegenerative Diseases, Institute for Translational Medicine, School of Basic Medical Sciences, Fujian Medical University, Fuzhou, Fujian 350122, China.

**Keywords:** Amyloid-β (Aβ), Death-associated protein kinase 1 (DAPK1), Phosphorylation, Tau, Oligomer

## Abstract

The aggregation of amyloid-β (Aβ) peptides into oligomers and fibrils is a key pathological feature of Alzheimer's disease (AD). An increasing amount of evidence suggests that oligomeric Aβ might be the major culprit responsible for various neuropathological changes in AD. Death-associated protein kinase 1 (DAPK1) is abnormally elevated in brains of AD patients and plays an important role in modulating tau homeostasis by regulating prolyl isomerase Pin1 phosphorylation. However, it remains elusive whether and how Aβ species influence the function of DAPK1, and whether this may further affect the function and phosphorylation of tau in neurons. Herein, we demonstrated that Aβ aggregates (both oligomers and fibrils) prepared from synthetic Aβ42 peptides were able to upregulate DAPK1 protein levels and thereby its function through heat shock protein 90 (HSP90)-mediated protein stabilization. DAPK1 activation not only caused neuronal apoptosis, but also phosphorylated Pin1 at the Ser71 residue, leading to tau accumulation and phosphorylation at multiple AD-related sites in primary neurons. Both DAPK1 knockout (KO) and the application of a specific DAPK1 inhibitor could effectively protect primary neurons against Aβ aggregate-induced cell death and tau dysregulation, corroborating the critical role of DAPK1 in mediating Aβ aggregation-induced neuronal damage. Our study suggests a mechanistic link between Aβ oligomerization and tau hyperphosphorylation mediated by DAPK1, and supports the role of DAPK1 as a promising target for early intervention in AD.

## Introduction

Alzheimer's disease (AD) is the leading cause of dementia, accounting for more than 60% of total dementia cases worldwide 1. Pathologically, AD is characterized by the deposition of amyloid-β (Aβ) peptides in the brain parenchyma and the accumulation of hyperphosphorylated tau proteins in the form of neurofibrillary tangles in neurons 1. Clinical evidence has suggested that the accumulation of Aβ in the brain is the earliest detectable event in the progression of AD, occurring about a decade prior to the onset of cognitive dysfunction 2. Similar to Aβ accumulation in the parenchyma, the content of tau proteins has also been found to increase in cerebrospinal fluid (CSF) about 10 years before symptom onset 2. The early initiation of both Aβ and tau accumulation in the disease course of AD highlights the importance of these two proteins as targets for disease diagnosis and intervention.

Aβ oligomers are important intermediates formed during the self-assembly of Aβ and cover a wide range of molecular weights and morphologies 3. For example, Lesné *et al*. found that Aβ dimers, trimers and Aβ*56 (~56 kDa) are present in human brains, and the contents of these oligomers increase gradually with age 4. These types of oligomers are small in size and are therefore regarded as low molecular weight (LMW) species. Yang *et al*. identified a different group of soluble Aβ aggregates with high molecular weight (HMW) from the brains of AD patients. These species range from ~150 to 600 kDa based on size exclusion chromatography analysis 5. Although Aβ oligomers remain low in abundance and metastable in the CSF, they are usually soluble and diffusible in solution and manifest high structural heterogeneity, making these species rather detrimental to neurons 3, 6. Aβ oligomers have been proven to trigger tau phosphorylation in neurons, resulting in microtubule dysfunction and neurite degeneration. For instance, Aβ oligomers may activate protein kinases such as glycogen synthase kinase-3β (GSK-3β), Ca^2+^-dependent calmodulin kinase IIα (CaMKIIα) and cyclin-dependent kinase 5 (CDK5) that are able to directly phosphorylate tau proteins at AD-related sites 7-9. The exacerbation of tau pathology by Aβ oligomers implicates a toxic interplay between these two pathological changes in the central nervous system (CNS).

Death-associated protein kinase 1 (DAPK1) is a Ca^2+^/calmodulin-dependent serine/threonine kinase. DAPK1 plays a key role in modulating cell death and autophagy and is intimately involved in the pathogenesis of tumors, ischemic brain injury and neurodegenerative diseases 10, 11. Several studies have suggested that DAPK1 might be a risk factor for sporadic AD. For example, our previous study revealed for the first time that the DAPK1 protein level in the hippocampus of AD cases is significantly higher than that of controls 12. DAPK1 upregulation in AD not only affects Aβ pathology by altering APP phosphorylation and processing, but also influences the function of tau through multiple pathways 13. DAPK1 activation phosphorylates Pin1 at the Ser71 residue and inactivates its isomerization capability, which stabilizes tau proteins and promotes tau phosphorylation in the brain 12, 14. In addition, DAPK1 may indirectly induce tau phosphorylation at the Ser262 residue via microtubule affinity regulating kinase family members, causing microtubule disruption and tau toxicity 15. In relation to this, Pei *et al*. further discovered that DAPK1 could also directly phosphorylate tau at the Ser262 residue by interacting with the microtubule repeat domain of tau proteins 16. These findings clearly substantiate a pivotal role of the DAPK1/Pin1 pathway in modulating tau function in neurons. Although Aβ oligomers are able to stimulate tau phosphorylation, it is unclear whether DAPK1 is involved in this process.

The protein stability of DAPK1 can be regulated by the ubiquitin-proteasome system by binding to heat shock protein 90 (HSP90) or DAPK-interacting protein 1 17, 18. HSP90 has a fundamental role in regulating cellular stress responses through maintaining protein homeostasis. HSP90 forms complexes with DAPK1 by recognizing specific amino acid residues in the kinase domain 18. The complexation not only contributes to DAPK1 stabilization, but also facilitates its activation 18. Importantly, studies have demonstrated that HSP90 protein levels are upregulated in brains of AD patients 19. HSP90 inhibitors not only attenuate tau and Aβ pathologies, but also promote the proteasomal degradation of DAPK1 18, 20. However, whether Aβ oligomers could regulate DAPK1 function through HSP90 remains to be studied.

In the present study, we aimed to clarify whether Aβ oligomers could modulate tau phosphorylation by influencing DAPK1 function. In detail, we reported that soluble Aβ aggregates stabilized the DAPK1 protein through HSP90, leading to subsequent Pin1 dysfunction by phosphorylating the Ser71 residue. This further caused tau accumulation and hyperphosphorylation at multiple AD-related sites, as well as caspase-3 dependent apoptosis in primary neurons. Genetic or chemical deactivation of DAPK1 protected neurons against Aβ species-induced neuronal apoptosis and tau dysregulation. Our study implies a mechanistic link between Aβ and tau pathologies via DAPK1.

## Materials and Methods

### Chemicals and reagents

Synthetic human Aβ (1-42) peptides (Aβ42) were purchased from Chinapeptide (Shanghai, China). The DAPK1-specific inhibitor (4Z)-4-(3-pyridylmethylene)-2-styryl-oxazol-5-one (C6) was acquired from Calbiochem (California, United States). The HSP90 inhibitor 17-allylamino-17-demethoxygeldanamycin (tanespimycin, 17-AAG) was from Macklin (Shanghai, China). DMSO, trypan blue powder, 1, 1, 1, 3, 3, 3-hexafluoro-isopropanol (HFIP), cycloheximide (CHX), poly-D-lysine and cytosine β-D-arabinofuranoside were purchased from Sigma-Aldrich (Missouri, United States). 3-(4, 5-Dimethylthiazol-2-yl)-2,5-diphenyltetrazolium bromide (MTT), nonfat milk powder and bovine serum albumin (BSA) were from Sangon (Shanghai, China). Hoechst 33342 solution was purchased from Beyotime (Shanghai, China). Fetal bovine serum (FBS), Dulbecco's modified Eagle's medium (DMEM), sodium pyruvate, GlutaMAX™ Supplement, Hank's balanced salt solution (HBSS), glucose solution, trypsin and penicillin-streptomycin were provided by Gibco (Texas, United States). Neurobasal medium and the B-27 supplement were obtained from BasalMedia (Shanghai, China). The anti-phospho-Ser71-Pin1 (pS71-Pin1) antibody was prepared and purified by immunizing rabbits with a peptide sequence (CSQSRRPSSWR) of Pin1 containing the pS71 residue (Abmart, Shanghai, China).

### Animals

Pregnant wild type (WT) C57BL/6 mice were purchased from Shanghai Laboratory Animal Research Center and were kept with food and water supply until the delivery of pups. The production of DAPK1 knockout (KO) mice (on a C57BL/6 background) was described previously 21. All mice were maintained in the SPF facility of Fujian Medical University with a 12/12 h light/dark cycle and *ad libitum* access to food. All animal experiments were approved by the Animal Ethics Committee of Fujian Medical University.

### Primary neuron culture

Primary neurons from WT and DAPK1 KO mice were prepared according to previous studies 22, 23, with minor modifications. Briefly, P0 pups were decapitated for cortical tissues. Cells were isolated and resuspended in DMEM supplemented with 10% FBS, 100 U/ml penicillin, 100 μg/ml streptomycin and sodium pyruvate. Cells were then plated onto poly-D-lysine-coated plates. After 4 h of culture at 37 °C and 5% CO_2_, the medium was replaced with neurobasal medium containing 2% B-27, GlutaMAX supplement, 100 U/ml penicillin and 100 μg/ml streptomycin. Cytosine-β-D-arabinofuranoside was added to the medium at 3 days *in vitro* (DIV3) to obtain neuronal-enriched cultures. Cells were maintained at 37 °C and 5% CO_2_ by replacing half of the medium with fresh neurobasal medium every two days. Cultures were used at DIV7-9 for experiments.

### Preparation and characterization of Aβ42 species

The preparation of Aβ42 species in different aggregation states was performed according to Stine *et al*. 24, 25. In brief, synthetic Aβ42 peptides (1 mg) were first dissolved in 250 μL of cold HFIP and incubated at room temperature overnight. HFIP was then removed using a lyophilizer (SP Scientific, Pennsylvania, United States). Afterward, Aβ42 peptides were reconstituted using DMSO to obtain a stock concentration of 5 mM. To prepare LMW Aβ species, the Aβ42 stock was diluted with cold PBS to a final concentration of 100 μM. The PBS solution was incubated at 4 °C for 24 h. To obtain HMW Aβ species, the stock was diluted using 10 mM HCl solution to 100 μM and was subsequently incubated at 37 °C for 24 h. The aggregation states of both samples were examined using tris-tricine gel (15%) electrophoresis and the 6E10 antibody (BioLegend, California, United States). To validate the presence of Aβ oligomers, we also conducted dot blot analysis using an anti-oligomer A11 antibody (Thermo Fisher, Massachusetts, United States). The preparation of Aβ42 species was frequently checked to maintain the quality of Aβ used in the experiments.

### MTT assay

To examine the cytotoxicity of Aβ42 species, primary neurons were seeded in poly-D-lysine-coated 96-well plates (5×10^4^ cells/well). At DIV7, cells were treated with different concentrations of HMW or LMW Aβ42 species (0.5, 2, 10 and 20 μM, equivalent to monomers) or vehicle for 24 h. MTT stock (5 mg/mL in PBS) was added into each well to a working concentration of 0.5 mg/mL. After 4 h of incubation at 37 °C and 5% CO_2_, the cell medium was removed, and DMSO at 100 μL/well was added to dissolve formazan crystals. The plate was shaken for 10 min and subjected to absorbance measurements at 570 nm and 630 nm using a microplate reader (Thermo Fisher). All samples were prepared in triplicate.

### Trypan blue staining

To quantify neuronal cell death caused by Aβ42 species, primary neurons were seeded in coated 48-well plates (5×10^4^ cells/well). At DIV7, cells were treated with different concentrations of LMW or HMW Aβ42 species or vehicle for 24 h. The trypan blue stock solution (0.4% in PBS) was diluted 10-fold using PBS. Trypan blue solution was added to the cells (300 μL/well), and the plate was incubated at room temperature for 5 min. The solution was replaced with PBS and cells were imaged under a microscope (Zeiss, Oberkochen, Germany). At least three different fields were recorded for the same sample. The images were analyzed using ImageJ software (version 1.50i, NIH, United States) to count live and dead cells.

### TUNEL assay

To further determine Aβ42-induced cell death, we carried out a TUNEL assay using an *in situ* cell death detection kit, TMR red (Roche, Indiana, United States), following the manufacturer's manual. Primary neurons (5×10^4^ cells/well) were seeded in 24-well plates containing poly-D-lysine-coated coverslips (EMS, Pennsylvania, United States). At DIV7, neurons were treated with various concentrations of LMW or HMW Aβ42 species or vehicle for 24 h. TUNEL reagents were added to each well after cell fixation and permeabilization, and were incubated with cells at 37 °C for 1 h. After washing with PBS, cells were incubated with Hoechst 33342 (1:2000 dilution) to stain nuclei. All samples were imaged using a fluorescence microscope (Zeiss). At least three different fields were recorded for each sample. The data were evaluated with ImageJ (version 1.50i) to quantify TUNEL-positive cells.

### Immunoblot analysis

Primary neurons were seeded in poly-D-lysine-coated 6-well plates at a density of 8×10^5^ cells/well. At DIV7, cells were treated with either Aβ42 species alone or together with inhibitors, as indicated in each experiment. After 24 h, the cells were harvested and lysed using radioimmunoprecipitation assay buffer (RIPA buffer) in the presence of protease and phosphatase inhibitor cocktails (Transgene, Beijing, China). The protein concentration was determined using a BCA protein assay kit (Beyotime). Protein samples (10-15 μg) were separated by SDS-PAGE and transferred to 0.45-μm polyvinylidene fluoride membranes (Millipore, Massachusetts, United States). Following transfer, the membranes were blocked with 5% BSA-TBST or 5% milk-TBST at room temperature for 1 h. The membranes were probed with primary antibodies and HRP-conjugated secondary antibodies. Target proteins were further detected using ECL chemiluminescent HRP substrate (Millipore) in a Bio-Rad Chemidoc imaging system (California, United States). All blots were analyzed using the ImageJ software (version 1.50i) to determine the optical densities. Data were normalized to the corresponding β-actin bands. The primary antibodies used in the study can be found in the Supplementary Information (Table S1).

### RNA extraction and qPCR

Whole RNA was isolated from primary neurons treated with Aβ species or vehicles using NucleoZOL (Macherey-Nagel, Dueren, Germany). The cDNA was first synthesized using HiScript II Q RT SuperMix (Vazyme, Nanjing, China). qPCR assays were then performed using ChamQ Universal SYBR qPCR Master Mix (Vazyme) in a QuantStudio Real-Time PCR system (Applied Biosystems, Massachusetts, United States). Data were analyzed with the comparative Ct (ΔΔCt) method by normalizing the value to the 18S ribosomal RNA (rRNA) or GAPDH level. The primer sequences used in the study were as follows: mouse DAPK1, forward 5'-GCACCCAAATGTCATCACCCT-3', reverse 5'-AAACAGCTCACCTCCTGCAAC-3'; 18S rRNA, forward 5'-TGTCTCAAAG-ATTAAGCCATGCA-3', reverse 5'-GCGACCAAAGGA-ACCATAACTG-3'; GAPDH, forward 5'-AGGTCGGTGTGAACGGATTTG-3', reverse 5'- TGTAGACCATG-TAGTTGAGGTCA-3'.

### DAPK1 protein stability assay

The protein stability of DAPK1 in primary neurons was measured by incubating neurons (DIV7) with CHX (10 μg/ml) in the absence or presence of Aβ42 species for indicated times. Samples were collected at 0, 3, 6 and 12 h after CHX addition, and were subjected to immunoblot analysis to probe the protein level of DAPK1.

### Statistical analysis

Statistical analyses were conducted using the GraphPad Prism software (version 8.3.0, GraphPad, California, United States). Data were collected from at least three independent cell cultures as indicated in the figure legends. No test for outliers was conducted. The Shapiro-Wilk test was used for normality test. All data are expressed as mean ± standard deviation (SD). The statistical significance was analyzed by either two-tailed unpaired *t*-test or one-way ANOVA followed by Tukey's *post hoc* test. *p* value < 0.05 was considered significant.

## Results

### Characterization of Aβ42 species

**Figure 1:**
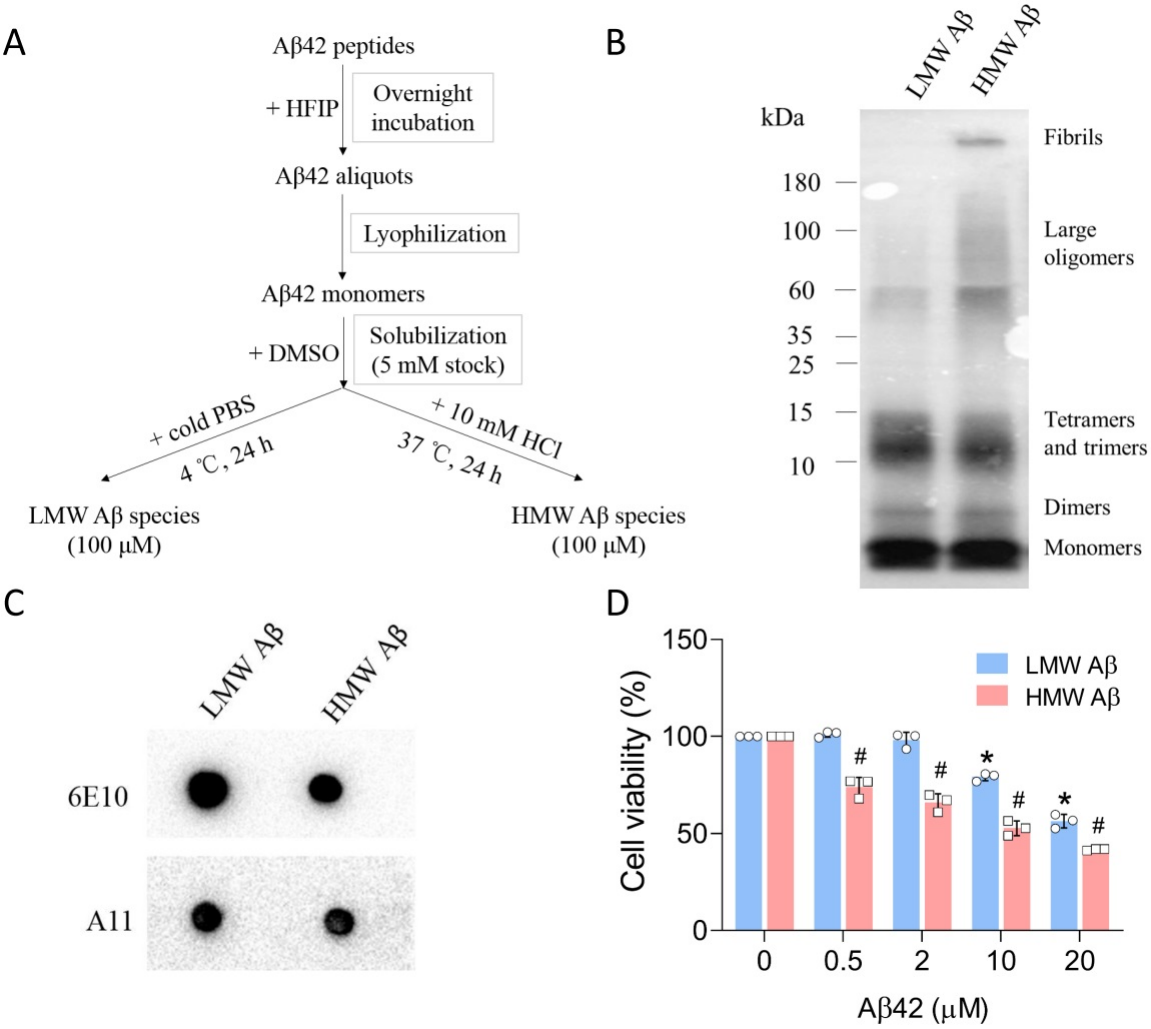
Preparation and characterization of Aβ42 species. (A) The protocol used for the preparation of LMW and HMW Aβ42 species from synthetic peptides. (B) Tris-tricine gel analysis of the size distribution of LMW and HMW Aβ42 species as probed by the 6E10 antibody. (C) Dot blot analysis of the presence of A11-positive species in both preparations. (D) MTT assay to measure the cytotoxicity of LMW and HMW Aβ42 species in mouse primary neurons. Data are expressed as the mean ± SD (* and ^#^, *p*< 0.05 vs. 0 μM).

The preparation of Aβ aggregates from synthetic Aβ42 peptides has been well described in the literature. By changing the incubation conditions (e.g., ionic strength and pH), Aβ species in different aggregation states can be formed. We followed the protocols developed by Stine *et al*. 25 and sought to prepare Aβ42 oligomers and fibrils (Figure 1A). The distribution of Aβ42 species was first examined using tris-tricine gel electrophoresis and the 6E10 antibody (Figure 1B). Under physiological pH and ionic strength and low temperature (4 *°*C), Aβ42 mainly formed LMW species between 10 and 15 kDa, corresponding to trimeric and tetrameric Aβ42 species. The dominant species below 10 kDa were monomers either from unaggregated Aβ materials or the dissociation of Aβ aggregates, or both. An additional band can be observed between the monomer and trimer bands, likely representing Aβ42 dimers (~9 kDa). When incubated at acidic pH and low ionic strength (10 mM HCl) at 37 *°*C, Aβ42 peptides also formed dimers, trimers and tetramers, as depicted by multiple bands below 15 kDa. Moreover, we observed a smear between 60 and 180 kDa, and a strong signal at the top of the membrane, suggesting the formation of large Aβ42 aggregates and fibrils with high molecular weight after incubation. The large assemblies were not visible in Aβ42 samples incubated at 4 *°*C and were termed HMW species. The size distributions of Aβ42 assemblies in our study were in accordance with those reported by Dahlgren *et al*. 26. To further validate the presence of pre-fibrillar oligomers in samples, we performed dot blot analysis using an oligomer-specific A11 antibody that recognizes Aβ species ranging from trimeric to ~75 kDa 27. As seen from the data (Figure 1C), Aβ42 aggregates from both preparations contained A11-positive species in solution. Since A11-positive oligomers are also present in brains of AD patients, we carried out MTT assays to evaluate the cytotoxicity of Aβ42 species prepared in our study (Figure 1D). Primary neurons were incubated with various concentrations (equivalent to monomers) of Aβ42 for 24 h. LMW Aβ42 species induced a significant decrease in cell viability at 10 μM, while HMW Aβ42 species markedly reduced cell viability at 0.5 μM and manifested a concentration-dependent toxic effect on primary neurons. Thus, we successfully prepared two types of Aβ42 species with partially overlapping compositions. These species contained physiologically relevant Aβ aggregates and were neurotoxic *in vitro*.

### Aβ42 species activate DAPK1 function in primary neurons

**Figure 2:**
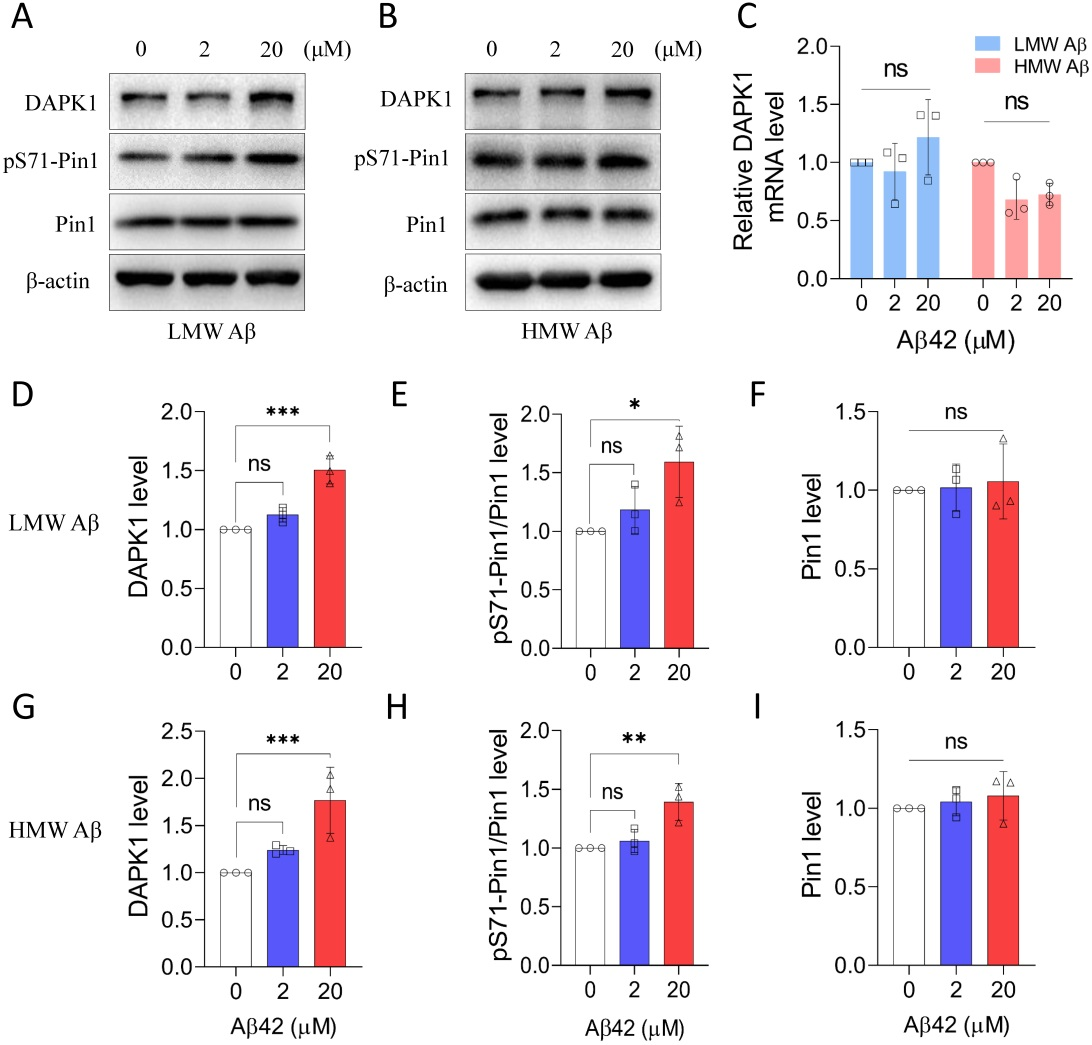
Aβ42 species upregulate DAPK1 protein levels and function. (A) and (B) Immunoblot analysis of DAPK1, pS71-Pin1 and Pin1 levels in primary neurons treated with different concentrations of LMW and HMW Aβ42 for 24 h. (C) Real-time PCR measurement of DAPK1 mRNA levels in WT primary neurons treated with different concentrations of LMW and HMW Aβ42 species. 18S ribosomal RNA was used as an internal control. The corresponding statistics for immunoblot analysis are shown in (D), (E) and (F) and (G), (H) and (I). β-actin was used as an internal control. Data are expressed as the mean ± SD (*p<0.05, **p<0.01, ***p<0.001, ns, not significant).

We next studied whether the prepared Aβ42 species were able to regulate the function of DAPK1 in primary neurons. Our previous research has demonstrated that DAPK1 could directly interact with Pin1 and phosphorylate its Ser71 residue 14. Therefore, we determined the level of pS71-Pin1 to monitor the enzymatic activity of DAPK1 upon Aβ42 treatment. Both LMW and HMW Aβ42 species were able to upregulate DAPK1 protein levels, especially at a concentration of 20 μM (Figure 2A, B, D and G), without affecting its mRNA level (Figure 2C and Figure S1). Corresponding to the elevation in DAPK1 expression, Pin1 phosphorylation at the Ser71 residue was also significantly increased in primary neurons incubated with 20 μM Aβ42 species (Figure 2A, B, E and H), whereas the total Pin1 level remained constant among neurons treated with or without Aβ42 species (Figure 2A, B, F and I). The increase in pS71-Pin1 levels clearly supported that Aβ42 species could enhance the function of DAPK1. Furthermore, our results indicated that Aβ aggregates upregulate DAPK1 expression through a transcription-independent mechanism. In addition, both Aβ preparations showed similar effects based on the quantification, suggesting that DAPK1 activation might be a common pathway in mediating Aβ aggregation-related cytotoxicity.

### DAPK1 activation mediates Aβ42 species-induced neuronal apoptosis

**Figure 3:**
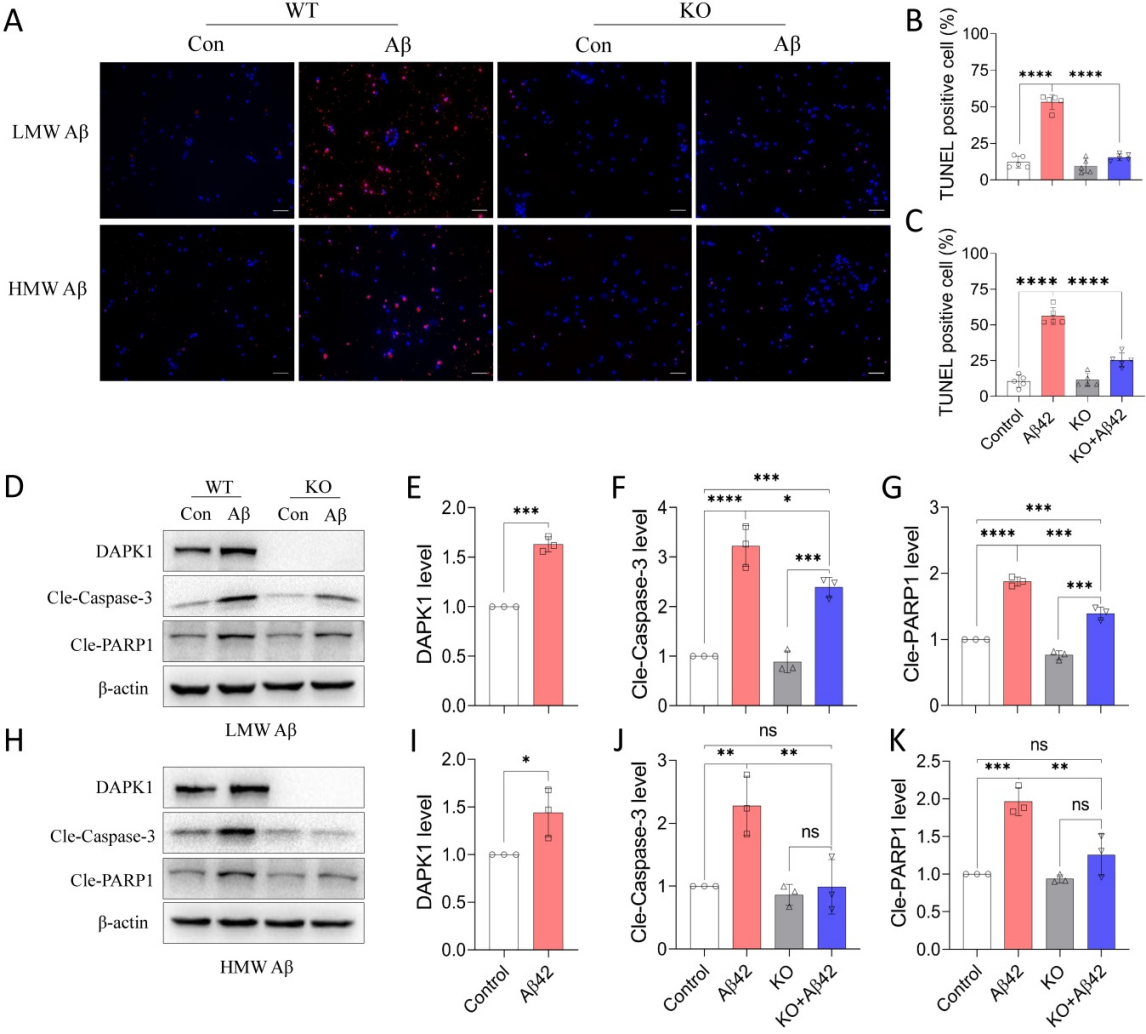
DAPK1 activation mediates Aβ42 species-induced neuronal apoptosis. (A) TUNEL assay showing cell death of WT or DAPK1 KO primary neurons treated with or without 20 μM Aβ42 species for 24 h. Blue staining indicates cell nuclei and magenta staining represents TUNEL-positive cells. Scale bar 50 μm. (B) and (C) Statistical analysis of the TUNEL assay for LMW and HMW Aβ species, respectively. (D) and (H) Immunoblot analysis of WT and DAPK1 KO primary neurons treated with LMW and HMW Aβ42 species (20 μM) for 24 h to detect DAPK1, cleaved caspase-3 and cleaved-PARP1 levels. (E), (F) and (G) and (I), (J) and (K) are the corresponding statistics. β-actin was used as an internal control. Data are expressed as the mean ± SD (*p<0.05, **p<0.01, ***p<0.001, ****p<0.0001, ns, not significant).

Since DAPK1 plays an important role in regulating cell survival under stress response, we further determined whether DAPK1 activation by Aβ42 species could result in neuronal cell death. We first applied a TUNEL assay to detect cell death in primary neurons. It is evident from the fluorescence imaging that TUNEL positive cells were increased dramatically with the addition of Aβ42 species (Figure 3A). Approximately 50% of cells died in WT neurons treated with 20 μM Aβ42 species, irrespective of the aggregation state (Figure 3B and C). In line with the TUNEL assay, trypan blue staining also showed that around 50% of WT neurons incorporated the dye in the presence of 20 μM Aβ42 species (Figure S2), pointing to substantial cell death caused by the treatment. However, none of the Aβ42 species were capable of triggering significant cell death in DAPK1 KO neurons, as shown in the TUNEL assay (Figure 3A, B and C), indicating a protective effect of DAPK1 ablation on Aβ-induced neuronal loss. To better understand how Aβ42 species lead to neuronal cell death, we evaluated apoptosis markers that are closely associated with DAPK1 activation. Consistent with the DAPK1 increase after Aβ42 species treatment (Figure 3D, E, H and I), levels of cleaved caspase-3 and cleaved poly-ADP-ribose polymerase-1 (PARP1) were markedly increased in neurons treated with 20 μM Aβ42 aggregates (Figure 3D and H), suggesting that Aβ42 induces neuronal cell death through caspase-3 dependent apoptosis. Moreover, we examined whether ablating DAPK1 protein was able to protect neurons against apoptosis caused by Aβ42 species. Although DAPK1 KO fully reversed HMW Aβ species-induced caspase-3 activation and PARP cleavage (Figure 3H, J and K), it only partially prevented LMW Aβ-induced apoptotic changes in primary neurons (Figure 3D, F and G). However, reduced cell death along with the decrease in cleaved caspase-3 and PARP1 levels in DAPK1 KO neurons treated with Aβ42 species substantiates the critical role of DAPK1 in mediating Aβ42 species-induced neuronal apoptosis.

### Aβ42 species promote tau accumulation and hyperphosphorylation by activating DAPK1

**Figure 4:**
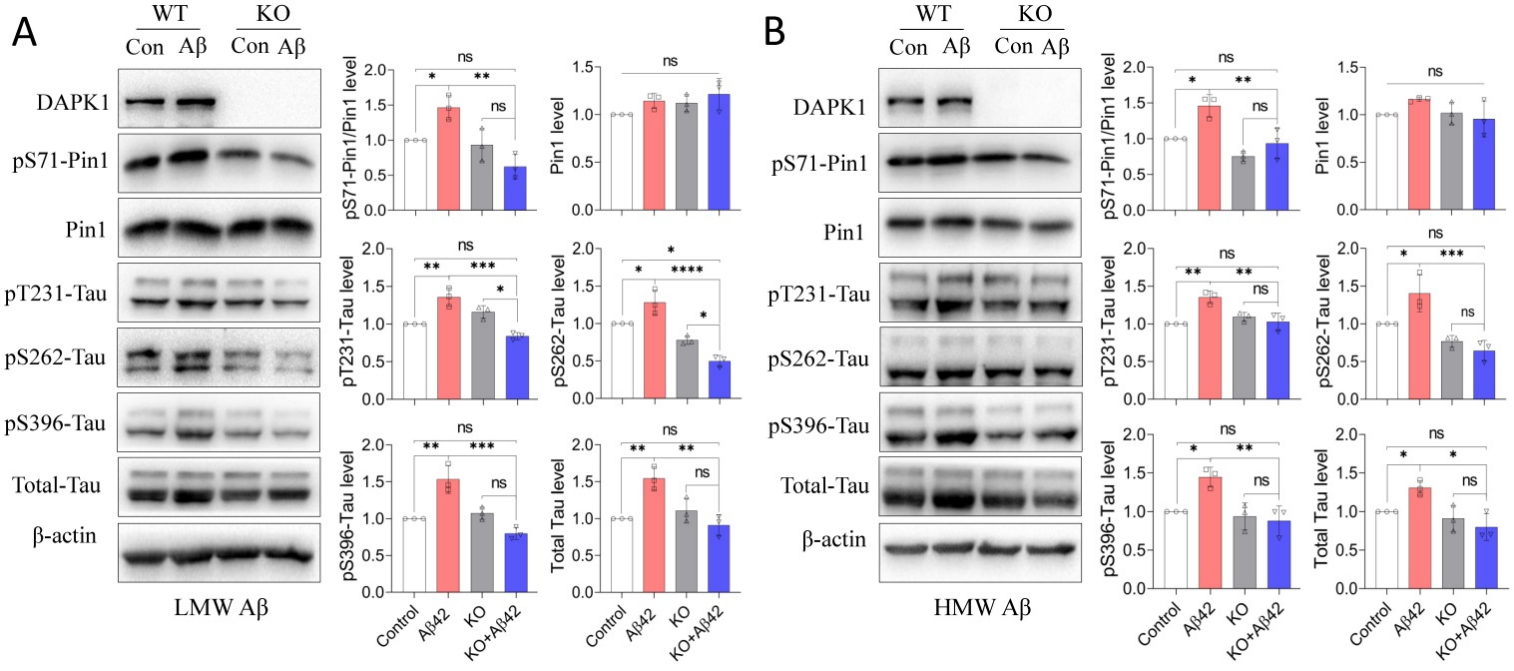
Aβ42 species induce tau accumulation and hyperphosphorylation via the DAPK1/Pin1 pathway. (A) and (B) Immunoblot analysis and statistics of WT and DAPK1 KO primary neurons treated with LMW or HMW Aβ42 species (20 μM) for 24 h to determine the levels of DAPK1, pS71-Pin1, Pin1, total tau and tau phosphorylation at the Thr231, Ser262 and Ser396 residues. β-actin was used as an internal control. Data are expressed as the mean ± SD (*p<0.05, **p<0.01, ***p<0.001, ****p<0.0001, ns, not significant).

The interplay between Aβ aggregation and tau dysregulation is essential for understanding the pathoetiology of AD, as well as for developing disease-modifying treatments. Previous studies have indicated that soluble Aβ species could not only promote tau phosphorylation by regulating protein kinases such as CDK5 and GSK-3β 28, but also enhance the intracellular aggregation and intercellular spreading of tau proteins 29, 30. Pin1 could regulate the protein stability of tau and its phosphorylation state, which are both crucial for maintaining the physiological function of tau in neurons 31. It has been shown that DAPK1 activation triggers aberrant tau phosphorylation in AD and stroke 12, 16, while whether DAPK1 and Pin1 also participate in Aβ species-induced tau dysregulation remains to be determined. We therefore first compared the phosphorylation of Pin1 at the Ser71 residue (pS71-Pin1) in WT and DAPK1 KO primary neurons after Aβ42 species treatment. As shown in figure 4, all neurons had comparable Pin1 levels. WT neurons treated with Aβ42 species exhibited a significant increase in pS71-Pin1 levels, while this increase was diminished in DAPK1 KO neurons exposed to Aβ42 (Figure 4A and B). Pin1 phosphorylation at Ser71 has been known to negatively regulate its substrate binding affinity and isomerization activity, thereby decelerating the conformational conversion of *cis*-ptau to *trans*-ptau, which is essential for maintaining tau homeostasis 32, 33. We further detected total and phospho-tau levels in WT and DAPK1 KO samples. Corresponding to the change in pS71-Pin1 levels, both the total tau and tau phosphorylation at AD-related sites, including Thr231, Ser262 and Ser396, were remarkably elevated in WT neurons incubated with Aβ42 species, whereas they were not affected by Aβ42 treatment in DAPK1 KO primary neurons (Figure 4A and B). This result confirmed our previous report that DAPK1 activation leads to abnormal tau accumulation by inactivating Pin1 function 12. Our findings also hinted that LMW and HMW Aβ42 species showed similar effects on tau accumulation and phosphorylation. These results reveal for the first time that DAPK1 activation indeed participates in Aβ42 species-induced tau dysregulation and support a mechanistic link between Aβ and tau pathologies in the progression of AD.

### The DAPK1-specific inhibitor C6 protects neurons against Aβ42 species-induced apoptosis and tau dysregulation

**Figure 5:**
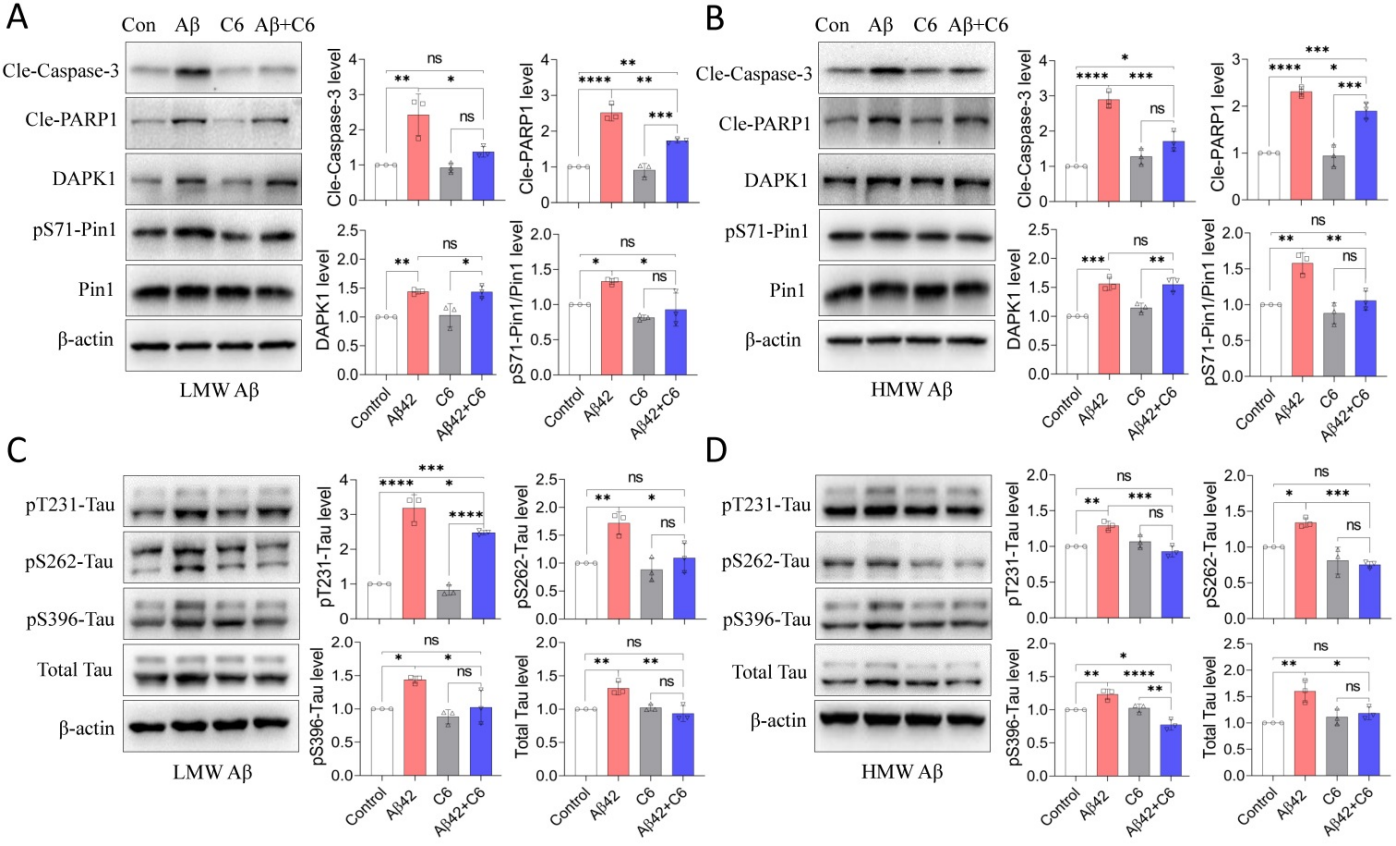
DAPK1 inhibition by C6 protects primary neurons against Aβ42 species-induced neuronal apoptosis and tau dysregulation. (A) and (B) Immunoblot analysis and statistics of cleaved caspase-3, cleaved PARP1 and DAPK1 function in WT primary neurons treated with 20 μM Aβ species alone or together with 2 μM C6 for 24 h. (C) and (D) Immunoblot analysis and statistics of total tau and tau phosphorylation at Thr231, Ser262 and Ser396 in WT primary neurons treated with Aβ species alone or together with C6 for 24 h. β-actin was used as an internal control. Data are expressed as the mean ± SD (*p<0.05, **p<0.01, ***p<0.001, ****p<0.0001, ns, not significant).

Because of the beneficial effect of DAPK1 KO against Aβ42 species-induced cell death and tau phosphorylation, we further investigated the impact of a DAPK1-specific inhibitor on Aβ42-induced neuronal damage to confirm the efficacy of DAPK1 inhibition in AD. The compound C6 (4-(pyridin-3-ylmethylene)oxazol-5(4H)-one) is a selective and potent inhibitor of DAPK1 and has been used as a potential treatment for ischemic brain injury in mouse models 34, 35. We first verified that 2 μM C6 treatment was able to suppress DAPK1 activity, as indicated by a significant increase in pSer308-DAPK1 levels (Figure S3). As shown in figure 5A and B, C6 treatment significantly attenuated Aβ42 species-induced Pin1 phosphorylation at Ser71 without affecting Pin1 levels, further confirming the inhibition of DAPK1 function by the compound. In parallel with DAPK1 inhibition, the activation of caspase-3 and the cleavage of PARP1 were significantly ameliorated by treatment with C6 in neurons exposed to Aβ42 species (Figure 5A and B), implicating an improvement in Aβ42-induced neuronal apoptosis following C6 treatment. The impact of C6 on Aβ42 species-induced tau accumulation and hyperphosphorylation was also assessed using an immunoblotting assay. The level of total tau protein in cells treated with both Aβ42 species and C6 was similar to that of the control samples (Figure 5C and D). In agreement with the reduction in total tau level, C6 treatment entirely reversed HMW Aβ42 species-induced tau phosphorylation at the Thr231, Ser262 and Ser396 residues (Figure 5D). DAPK1 inhibition by C6 was also capable of fully blocking the elevation of pS262 and pS396-tau caused by LMW Aβ42; however, the increase in the pT231-tau level was only partially reduced by the compound compared with neurons treated with Aβ42 alone (*p* < 0.05) (Figure 5C). These results again indicate that inhibiting DAPK1 activity confers protective effects on Aβ aggregation-induced neuronal damage. Together with findings from DAPK1 KO primary neurons, inhibition of DAPK1 function provides significant protection against Aβ42 aggregation-induced cell apoptosis and tau dysregulation, further corroborating the pivotal role of DAPK1 in modulating the key pathologies of AD.

### Aβ42 species increase the protein stability of DAPK1 by modulating HSP90 function

**Figure 6:**
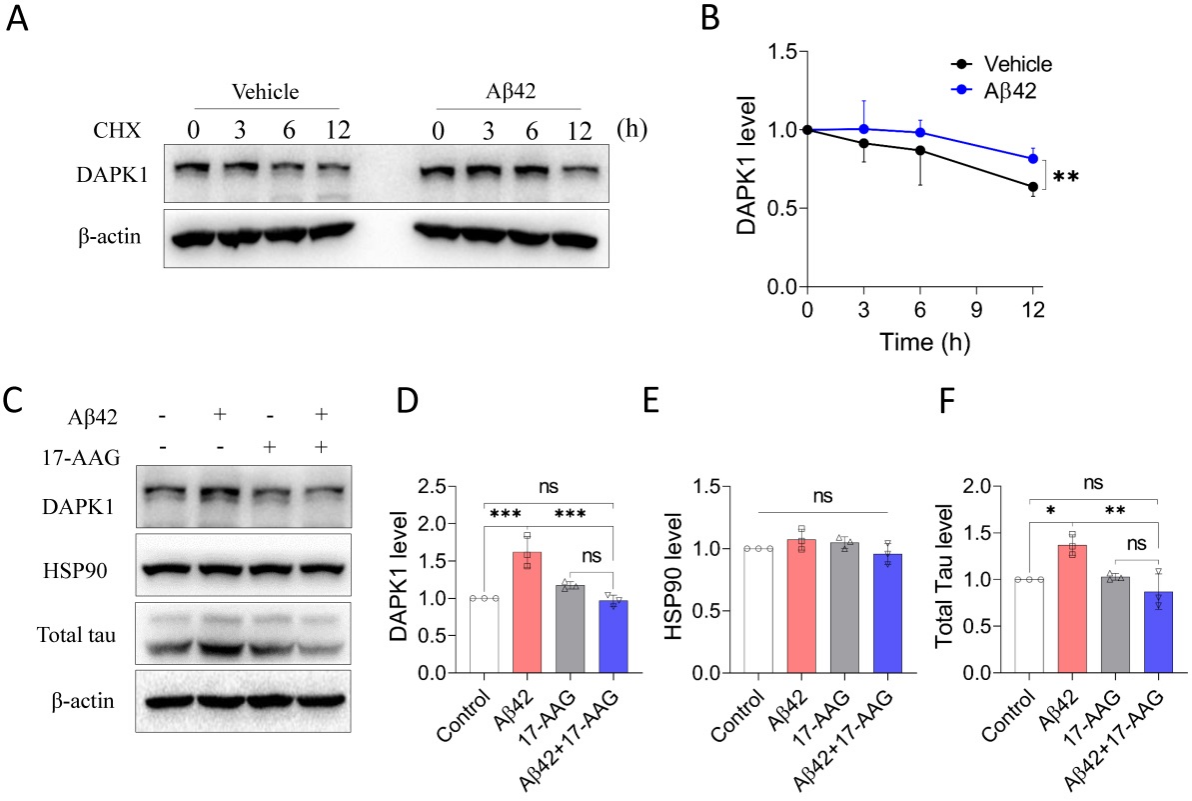
Aβ42 species stabilize DAPK1 protein by modulating HSP90 function. (A) and (B) CHX assay to determine DAPK1 protein levels in WT primary neurons treated with vehicle or 20 μM Aβ42 species for 0, 3, 6 and 12 h. The concentration of CHX was 10 μg/ml. (C) Immunoblot analysis of WT primary neurons treated with Aβ42 species alone or together with the HSP90 inhibitor 17-AAG (200 nM) for 24 h to evaluate DAPK1, HSP90 and total tau levels. (D), (E) and (F) are the corresponding statistics. β-actin was used as an internal control. Data are expressed as the mean ± SD (*p<0.05, **p<0.01, ***p<0.001, ns, not significant).

**Figure 7:**
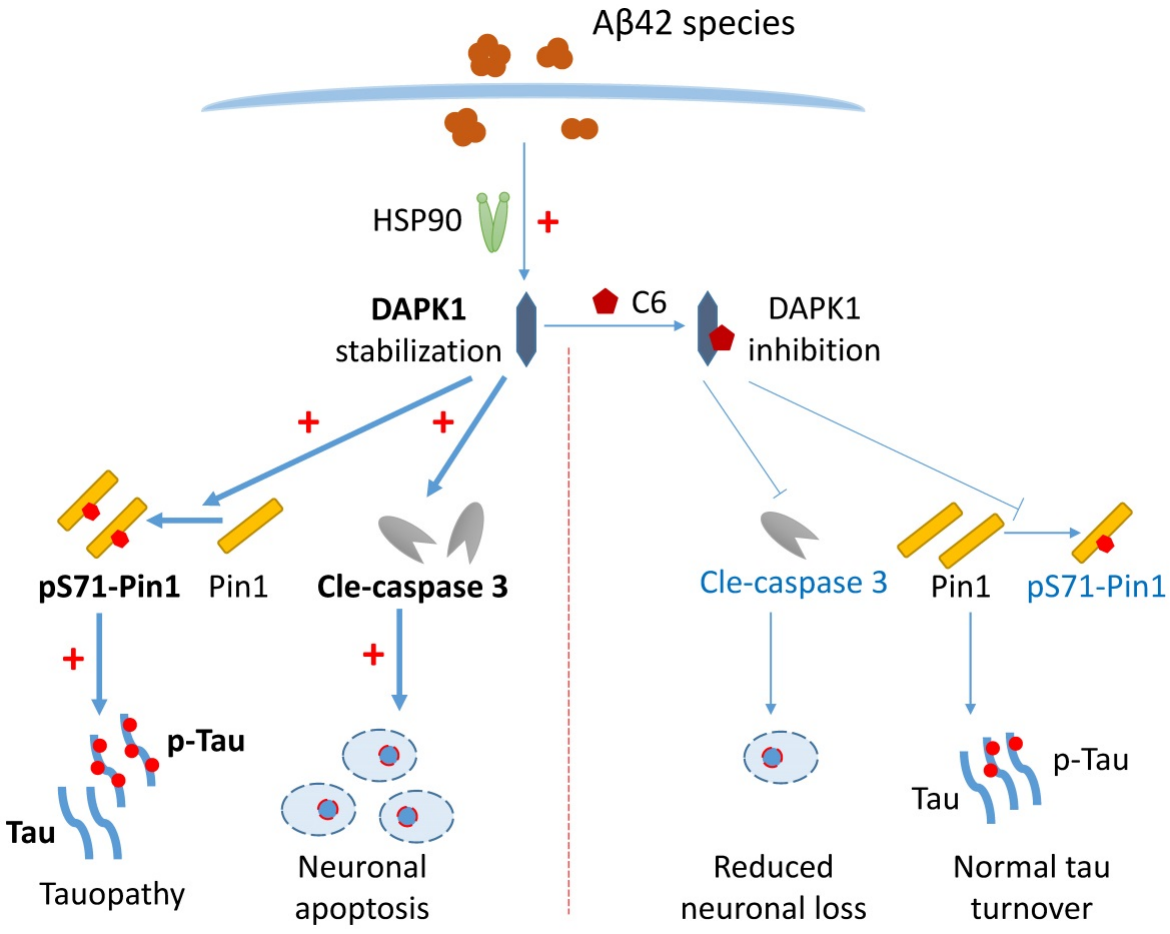
Schematic showing how Aβ42 species regulate DAPK1 and neuronal functions. The presence of Aβ42 species leads to DAPK1 stabilization and activation through the molecular chaperone HSP90. DAPK1 then inactivates Pin1 by phosphorylating its Ser71 residue, resulting in tau accumulation and hyperphosphorylation in neurons. In addition, DAPK1 activation also induces neuronal apoptosis by a caspase-3 dependent mechanism. DAPK1 inhibition by C6 protects neurons from Aβ-induced abnormal tau accumulation and mitigates neuronal loss. Upregulated proteins under AD conditions are shown in bold (left panel), and downregulated proteins caused by DAPK1 inhibition are depicted in blue (right panel).

Because DAPK1 mRNA levels were not significantly altered by Aβ42 species (Figure 2C), to examine the regulatory effect of Aβ on DAPK1 expression, we then assessed the protein stability of DAPK1 in neurons following Aβ42 species treatment using the CHX assay. According to figure 6A and B, DAPK1 protein was degraded over time in the presence of CHX treatment, while primary neurons incubated with Aβ42 had a slower DAPK1 degradation rate compared with neurons treated with vehicle. At 12 h, the level of DAPK1 in the Aβ42 group was also significantly higher than that in the vehicle group. Therefore, we concluded that the upregulation of DAPK1 after Aβ42 treatment is likely a result of reduced DAPK1 degradation. Due to the close association between HSP90 and DAPK1 stability and the elevation of HSP90 levels in the brains of AD patients 10, 19, we hypothesized that Aβ42 induced DAPK1 upregulation might be mediated by HSP90. To prove this, we coincubated primary neurons with Aβ42 species and tanespimycin (17-AAG), a potent HSP90 inhibitor, and detected whether Aβ42 was still able to increase DAPK1 protein levels. As evident from figure 6C and D, 17-AAG alone had a limited effect on DAPK1 expression, while it could significantly block Aβ42 species-induced DAPK1 elevation, implicating a causative role of HSP90 in modulating Aβ42-stimulated DAPK1 upregulation. Interestingly, we did not observe any change in HSP90 protein levels among all samples (Figure 6C and E); however, it has been suggested that the cellular function of HSP90 might be activated by Aβ aggregates as a result of the unfolded protein response 36. Along with DAPK1 reduction, 17-AAG also robustly decreased Aβ42-induced tau accumulation (Figure 6C and F), further verifying the function of DAPK1 in connecting Aβ aggregation and tau pathologies. Thus, our results indicate that the activation of HSP90 function by Aβ aggregates contributes to DAPK1 stabilization, whereby Aβ species further lead to neuronal apoptosis and aberrant tau accumulation (Figure 7).

## Discussion

Studies have revealed that Aβ aggregation and tau hyperphosphorylation in the CNS may occur decades before significant cognitive dysfunction, whereas how the two pathological features of AD affect each other has not been fully resolved. In the present study, we used primary neurons as *in vitro* models to study effects of LMW and HMW Aβ species on neuronal survival and tau phosphorylation. We discovered that Aβ42 aggregates activate DAPK1 function through HSP90-mediated DAPK1 stabilization, which then leads to Pin1 inactivation in neurons by phosphorylating the Ser71 residue. Furthermore, Pin1 inhibition results in the accumulation of hyperphosphorylated tau and total tau proteins in primary neurons. In addition, the upregulation of DAPK1 also contributes to caspase-3 dependent neuronal apoptosis. Inhibiting DAPK1 could significantly prevent Aβ aggregation-induced cell death and tau dysregulation. Our data illustrate that DAPK1 might be a key regulator of the crosstalk between Aβ aggregation and tau dysfunction in AD.

Recent studies have emphasized that soluble Aβ oligomers are the main contributor to neuronal dysfunction in the brain, causing extensive neurotoxic effects including neuroinflammation, oxidative stress and synaptic damage 6. Despite the fact that the physiological concentration of Aβ is well below the micromolar range 29, Aβ oligomers have been shown to excessively accumulate in synaptic clefts and periplaque regions 37, and might reach micromolar concentrations 38. The viability assay showed that both LMW and HMW Aβ species are cytotoxic in a concentration-dependent manner, suggesting that our experimental conditions are still relevant to physiological conditions. Research has demonstrated that the neurotoxicity of Aβ aggregates might be determined by the size and structural properties of these species. Small Aβ species formed at the early stage of aggregation can efficiently disrupt cell membrane integrity, while large Aβ species produced at a later stage of aggregation are prone to triggering neuroinflammatory responses 39. In accordance with previous studies 26, LMW and HMW Aβ42 species both contained small Aβ oligomers below 15 kDa. HMW Aβ species are more heterogeneous than LMW Aβ as they also have some larger Aβ aggregates and fibrillar structures in solution. The partial overlap in the size distribution between LMW and HMW Aβ species may explain similar outcomes of these two species in causing neuronal apoptosis and tau dysregulation. The analogous effects caused by LMW and HMW Aβ species implicate that different Aβ species share common pathological features in the CNS. However, this could also be attributed to a neuron-enriched condition used for assessing the effect of Aβ species, as large Aβ aggregates such as protofibrils tend to interact with glial cells rather than with neurons 39. Nevertheless, we were not able to dissect the distinct functions of oligomeric and fibrillar Aβ in regulating DAPK1 and tau under our experimental setup. Further studies are needed to characterize in detail how different Aβ species influence neuronal functions.

The activation or upregulation of DAPK1 in the brain has been reported to contribute to synaptic loss, Aβ generation and tau hyperphosphorylation in AD 12, 40, 41, while the molecular mechanism mediating DAPK1 upregulation in AD has not been revealed. A subsequent study found that Aβ25-35 was able to activate DAPK1 through cathepsin B-mediated regulation 42, which seems to contradict previous research showing that cathepsin B deficiency increases the protein level of DAPK1 43. The regulation of DAPK1 function in cells has been recognized to be dominated by posttranslational mechanisms, such as proteasome- or lysosome-mediated protein degradation 43. As an important executor of cellular proteostasis, HSP90 inhibition not only induces the degradation of DAPK1, but also significantly increases the level of DAPK1 phosphorylation at Ser308, synergistically leading to the suppression of DAPK1 function 10. More importantly, HSP90 is upregulated and shows colocalization with Aβ in brains of AD patients 19. We observed that both Aβ42 preparations elevated DAPK1 protein levels in neurons without affecting its mRNA level, but through enhancing the protein stability of DAPK1. The application of the HSP90 inhibitor 17-AAG blocked DAPK1 elevation and tau accumulation induced by Aβ, indicating that HSP90 activation may act upstream of DAPK1 upregulation in this context. In light of the early onset of abnormal Aβ accumulation in the progression of AD, we propose that DAPK1 upregulation induced by Aβ aggregation might also be an early event in the disease course of AD. Since the activation of DAPK1 function could contribute to the amyloidogenic processing of APP and the hyperphosphorylation of tau 44, DAPK1 may serve as an important therapeutic target for the simultaneous control of both amyloidosis and tauopathy in AD.

Pin1 is a well-known phospho-specific prolyl isomerase that catalyzes the *cis-trans* conformational change in pSer/Thr-Pro bonds of target proteins. Tau phosphorylation at Thr231 (pT231-tau) is the only phosphorylation epitope in tau that can be recognized and isomerized by Pin1 33. *Trans*-ptau proteins possess normal microtubule binding affinity and can be dephosphorylated by protein phosphatases in neurons. They are also prone to undergo protein degradation and are therefore correlated with low neurodegenerative phenotypes 33. In contrast, *cis*-ptau has completely opposite properties to *trans*-ptau and is extremely toxic to neurons 33. Pin1 inactivation greatly reduces the degradation of *cis*-ptau, blocking the normal turnover of tau proteins in neurons as a consequence. Thus, the activity of Pin1 has a profound impact on intracellular tau homeostasis by balancing the conformational change of pT231-tau proteins. Consistent with our previous findings about DAPK1 and Pin1 14, we found that the activation of DAPK1 by Aβ42 species also led to Pin1 inactivation through Ser71 phosphorylation, which agrees with Sultana *et al*.'s finding that AD patients have a significant loss of Pin1 activity in the hippocampus compared with healthy controls 45. Alternatively, Aβ could modulate Pin1 function through promoting its dephosphorylation. Stallings *et al*. demonstrated that soluble Aβ42 species could increase calcineurin-mediated dephosphorylation of Pin1 at the Ser111 residue, thus downregulating its isomerase activity and eventually leading to dendritic spine loss in the brain 46. However, the impact of Aβ42 species on Pin1 activity might be time-dependent, as Bulbarelli *et al*. reported that Aβ42 oligomers may activate Pin1 by favoring the dephosphorylation of its Ser16 residue at the early stage of the treatment (e.g., 3-8 h). Pin1 function returned to the basal level after sustained Aβ treatment (e.g., 24 h). The transient activation of Pin1 by Aβ could improve the dephosphorylation of pT231-tau, thereby reducing the overall phosphorylation of tau proteins in neurons 47. This study also discovered that Aβ fibrils had limited effects on Pin1 function, which is different from our results. We speculate that the difference might be related to the different properties of Aβ species derived from the preparation protocols. It should also be noted that the function of GSK-3β and CDK5, the major tau-phosphorylating kinases in AD, was not significantly altered by Aβ species (Figure S4), further supporting the crucial role of DAPK1 in the crosstalk between Aβ and tau pathologies.

DAPK1 is widely accepted as an important regulator of neuronal cell death in a plethora of diseases including ischemic stroke, Parkinson's disease and AD 48. For instance, DAPK1 has been shown to directly bind to extrasynaptic N-methyl-D-aspartate receptors (NR2B) and phosphorylate its Ser1303 residue 49. Upon phosphorylation by DAPK1, NR2B increases its conductance toward Ca^2+^, triggering intracellular Ca^2+^ overload that promotes neuronal cell death 49. Furthermore, DAPK1 could also couple with p53 and phosphorylate its Ser23 residue. Phosphorylated p53 proteins can either translocate into the nucleus, where they can initiate proapoptotic gene transcription, or attach to the mitochondrial matrix to induce necrosis 50. You *et al*. also revealed that DAPK1 is capable of binding to and phosphorylating N-myc downstream-regulated gene 2 (NDRG2). DAPK1-induced NDRG2 phosphorylation at Ser350 promotes cell death through a caspase-3 dependent mechanism 51. We found that DAPK1 KO or inhibition by C6 was able to alleviate neuronal apoptosis induced by both LMW and HMW Aβ42 species by reducing caspase-3 activation and PARP1 cleavage, suggesting that DAPK1 acts as a common upstream regulator of Aβ42 aggregation-induced apoptosis. Pin1 is also a crucial player in modulating cell fate as it regulates the isomerization of a variety of phosphoproteins that are essential for the cell cycle, differentiation and cell survival 32. The function of Pin1 in programmed cell death is defined by its cellular context. On the one hand, Pin1 may promote neuronal apoptosis by disrupting mitochondrial function or by activating the caspase-3 pathway in developing neurons 52. On the other hand, Pin1 may also exert antiapoptotic effects by stabilizing proteins of the Bcl-2 family in oligodendrocytes 52. Although we did not have evidence to show that Pin1 inactivation by Aβ-induced DAPK1 upregulation played a direct role in neuronal apoptosis, the recovery of Pin1 function by DAPK1 inhibition correlated with the improvement in neuronal cell death. In contrast with Duan *et al*.'s report showing that DAPK1-mediated tau phosphorylation could counteract kinase-induced apoptosis in cell lines 53, the increased tau phosphorylation did not improve neuronal apoptosis in our study. We speculate that this is because Aβ species are capable of causing cell death through different mechanisms 54. This could also explain why inhibiting DAPK1 alone might be unable to completely reverse neuronal loss induced by Aβ. However, the significant reduction in apoptosis markers following DAPK1 inhibition implies that the DAPK1/Pin1 pathway could be a potential target for early intervention in Aβ species-induced cellular damage.

In summary, our results show that Aβ aggregates upregulate the protein level of DAPK1 through HSP90-mediated stabilization. The activation of DAPK1 subsequently results in a loss of function of Pin1 in primary neurons, leading to the abnormal accumulation of both total and hyperphosphorylated tau proteins (Figure 7). Additionally, DAPK1 upregulation by Aβ aggregates also contributes to neuronal loss via a caspase-3 dependent mechanism, while its inhibition mitigates Aβ aggregation-induced neuronal apoptosis and tau dysregulation (Figure 7). Our findings have uncovered a new mechanism through which different Aβ aggregates evoke abnormal tau accumulation in neurons, and suggest that targeting the DAPK1/Pin1 pathway could be a useful strategy for the intervention in Aβ aggregation-induced neuropathological changes throughout the progression of AD.

## Supplementary Material

Supplementary figures and table.

## Abbreviations

Aβ: Amyloid-β
AD: Alzheimer's disease
DAPK1: Death-associated protein kinase 1
CSF: Cerebrospinal fluid
LMW: Low molecular weight
HMW: High molecular weight
GSK-3β: Glycogen synthase kinase-3β
CDK5: Cyclin-dependent kinase 5
HSP90: Heat shock protein 90
PARP1: Poly-ADP-ribose polymerase-1
LOAD: Late onset Alzheimer's disease
