# Supplementary material for: Death-associated protein kinase 1 mediates Aβ42 aggregation-induced neuronal apoptosis and tau dysregulation in Alzheimer's disease
Source: Int J Biol Sci. 2022 Jan 1;18(2):693–706. doi: 10.7150/ijbs.66760 (PMC8741852; doi:10.7150/ijbs.66760)
Supplement: Supplementary file 1 — Supplementary figures and table. [file ijbsv18p0693s1.pdf]

Supplementary Materials

Zhang et al.

Death-associated protein kinase 1 mediates Aβ42 aggregation-induced neuronal apoptosis and tau dysregulation in Alzheimer's disease

Table S1. Information of primary antibodies used in the present study.

Antibody	Dilution	Source	Identifier
Mouse anti-β-actin	1:40000	Sigma-Aldrich	A5441
Mouse anti-DAPK1	1:1500	Sigma-Aldrich	D2178
Mouse anti-phospho-DAPK1 (Ser308)	1:2000	Sigma-Aldrich	D4941
Rabbit anti-Pin1	1:4000	Proteintech	10495-1-AP
Rabbit anti-pS71-Pin1	1:200	Abmart	
Rabbit anti-Oligomer A11	1:3000	Thermo Fisher Scientific	AHB0052
Mouse anti-β-Amyloid, 1-16 (clone 6E10)	1:6000	BioLegend	SIG-39320
Rabbit anti-Cleaved Caspase-3 (Asp175)	1:500	Cell Signaling Technology	9661
Rabbit anti-PARP1	1:1000	Cell Signaling Technology	9532
Mouse anti-Tau46	1:6000	Cell Signaling Technology	4019
Rabbit anti-pT231-Tau	1:6000	Abcam	ab151559
Rabbit anti-pS262-Tau	1:2000	Invitrogen	44-750G
Rabbit anti-pS396-Tau	1:5000	Anaspec	AS-54977
Rabbit anti-GSK-3α/β	1:2000	Cell Signaling Technology	5676
Rabbit anti-phospho-GSK-3β (Ser9)	1:2000	Cell Signaling Technology	5558
Rabbit anti-CDK5	1:2000	Cell Signaling Technology	2506
Rabbit anti-p35/25	1:1000	Cell Signaling Technology	2680

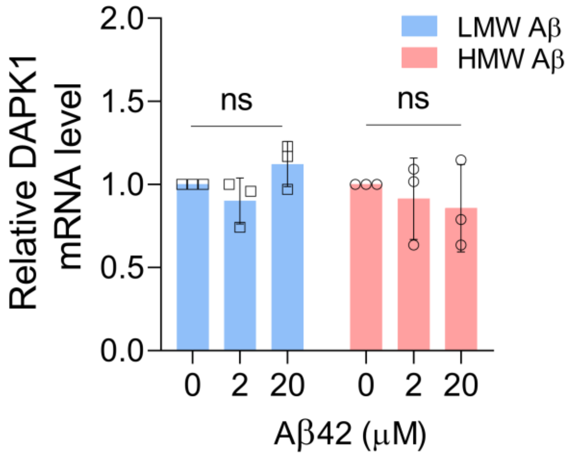

Figure S1. DAPK1 mRNA levels as determined by qPCR assay using GAPDH as a control. WT primary neurons were treated with different concentrations of Aβ42 species for 24 h. Samples were subjected to real-time PCR assay using GAPDH as an internal control (ns, not significant).

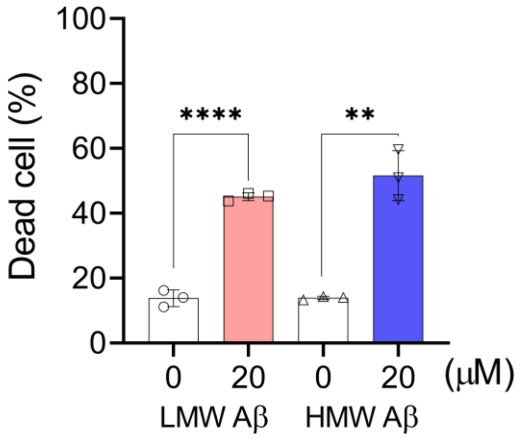

Figure S2. Trypan blue staining of WT primary neurons incubated with or without Aβ42 species for 24 h (**, p<0.01; ****,p<0.0001).

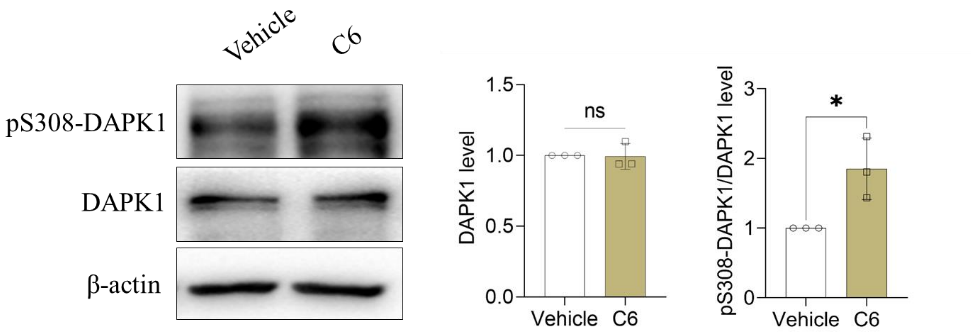

Figure S3. Detection of the inhibitory effect of C6 on DAPK1 activity as measured by its autophosphorylation at Ser308 residue. WT primary neurons were treated with vehicle or 2 μM C6 for 24 h. The level of pSer308-DAPK1 was determined to reflect the activity of DAPK1 (*, p<0.05, ns, not significant).

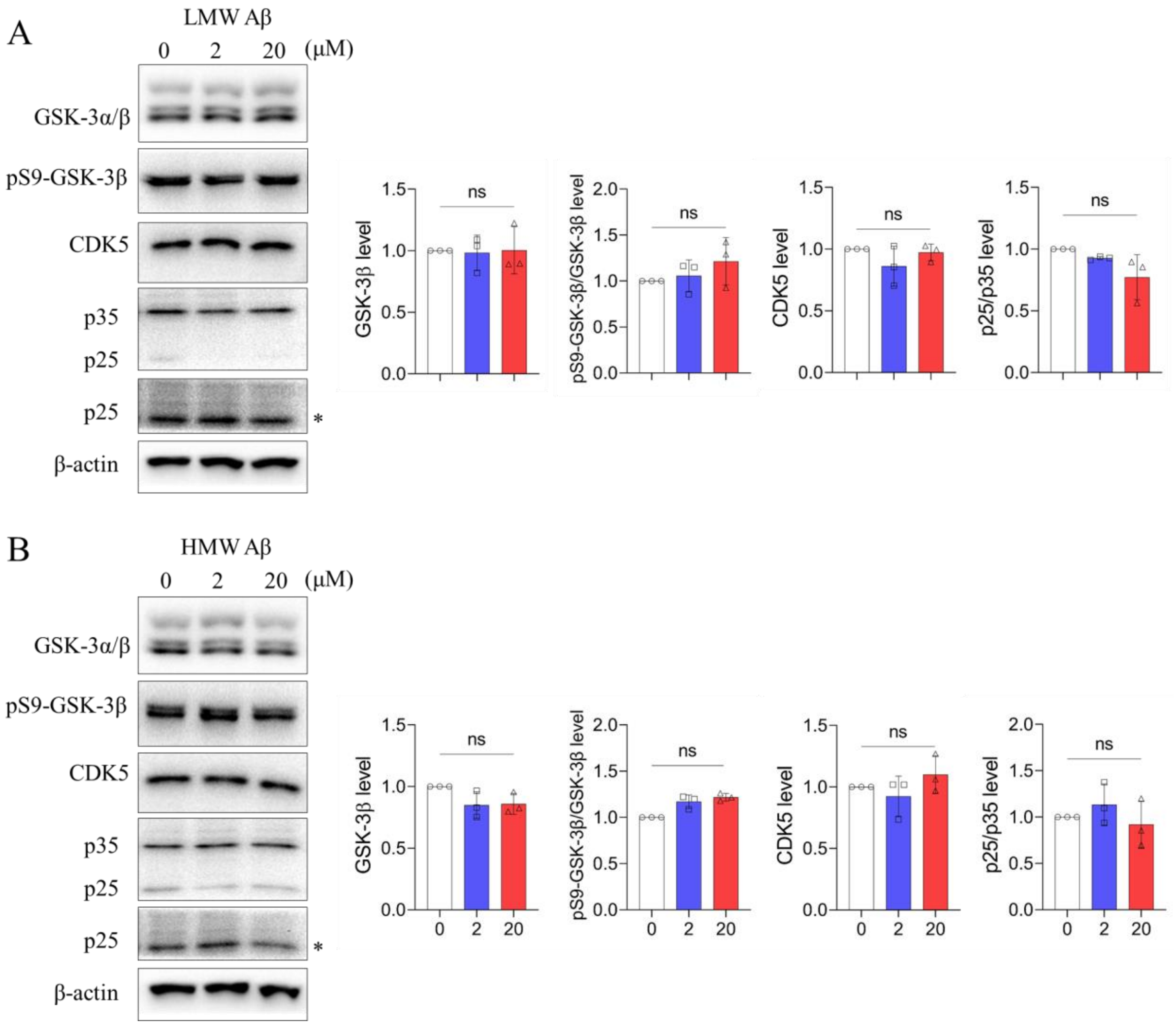

Figure S4. Analysis of the influence of Aβ42 species treatment on the function of GSK-3β and CDK5 in neurons. WT primary neurons were incubated with various concentrations of LMW (A) or HMW (B) Aβ42 species for 24 h. The expression as well as the function of GSK-3β and CDK5 was detected using immunoblot analysis. The function of GSK-3β was monitored by the ratio of pSer9-GSK-3β/GSK-3β, and the activity of CDK5 was determined by p25/p35 ratio (ns, not significant; * indicates longer exposure of the p25 band).
